# Manifold Feature Fusion with Dynamical Feature Selection for Cross-Subject Emotion Recognition

**DOI:** 10.3390/brainsci11111392

**Published:** 2021-10-23

**Authors:** Yue Hua, Xiaolong Zhong, Bingxue Zhang, Zhong Yin, Jianhua Zhang

**Affiliations:** 1 Engineering Research Center of Optical Instrument and System, Ministry of Education, Shanghai Key Lab of Modern Optical System, University of Shanghai for Science and Technology, Shanghai 200093, China; 192550430@st.usst.edu.cn (Y.H.); 182560463@st.usst.edu.cn (X.Z.); 2 School of Optical-Electrical and Computer Engineering, University of Shanghai for Science and Technology, Shanghai 200093, China; zhangbingxue@usst.edu.cn; 3 OsloMet Artificial Intelligence Lab, Department of Computer Science, Oslo Metropolitan University, N-0130 Oslo, Norway; jianhuaz@oslomet.no

**Keywords:** emotion recognition, electroencephalography, machine learning, feature selection, transfer learning

## Abstract

Affective computing systems can decode cortical activities to facilitate emotional human–computer interaction. However, personalities exist in neurophysiological responses among different users of the brain–computer interface leads to a difficulty for designing a generic emotion recognizer that is adaptable to a novel individual. It thus brings an obstacle to achieve cross-subject emotion recognition (ER). To tackle this issue, in this study we propose a novel feature selection method, manifold feature fusion and dynamical feature selection (MF-DFS), under transfer learning principle to determine generalizable features that are stably sensitive to emotional variations. The MF-DFS framework takes the advantages of local geometrical information feature selection, domain adaptation based manifold learning, and dynamical feature selection to enhance the accuracy of the ER system. Based on three public databases, DEAP, MAHNOB-HCI and SEED, the performance of the MF-DFS is validated according to the leave-one-subject-out paradigm under two types of electroencephalography features. By defining three emotional classes of each affective dimension, the accuracy of the MF-DFS-based ER classifier is achieved at 0.50–0.48 (DEAP) and 0.46–0.50 (MAHNOBHCI) for arousal and valence emotional dimensions, respectively. For the SEED database, it achieves 0.40 for the valence dimension. The corresponding accuracy is significantly superior to several classical feature selection methods on multiple machine learning models.

## 1. Introduction

Human emotions play an important role in conveying information about human–computer interaction and have a capability to indirectly reflect anxiety, stress and ability of cognition, communication and decision-making. With the wide application of machine learning methods in human-centered systems, emotion recognition has attracted much attention. Specifically, identified human’s emotions can be used as feedback to provide better service in medical care devices, recommender systems, and information retrieval engines [1]. It enhances the user experience and satisfaction level and leads to harmonious interactions between human and machine agents. Affective computing technique plays an important role in a wide range of applications in the domain of human-machine interactions. Thakur et al. designed a framework for human behavior monitoring [2]. Based on the data of human activities of daily living, multimodal components of user interactions were analyzed. The human behavioral patterns and their relationships with the dynamic contextual and spatial features of the environment functionalities were found to be significant. Machot et al. designed an affective computing module to support driver assistance systems [3]. They used Bayesian quadratic discriminating classifier to recognized driver’s emotional states that can help increase the performance of the transportation task. Affective computing can be also applied to understand behaviors of the digital society [4]. It quantified the collective emotions by means of fractal analysis and the topology of social networks. In [5], the social emotion was detected to predict the most appropriated songs to be played in a certain circumstance. In the domain of computer games, assessing player’s emotions with physiological signals in real time can maintain the engagement degree by properly adapting the game difficulty [6].

Human emotions can be explicitly reflected by the information conveyed from speech, facial expressions, gestures, and/or multimodalities of human behaviors. These external indicators possess an advantage of the data accessibility due to the use of noninvasive sensors, e.g., cameras and microphones. However, it is possible that people may hide their intrinsic emotional states when interacting with machine and/or computer agents [7]. The potential reason can be a common viewpoint on classical human-machine systems in which the machine is unable to properly understand human affective behaviors. On the other hand, affective states are closely related to the activities of human subsystems, e.g., the central nervous system, peripheral nervous system and somatic nervous system. Therefore, it is feasible to use physiological signals acquired by neuroimaging techniques as objective measurement to quantify emotion variations. For instance, the electroencephalography has a capability to scan multiple cortical regions that are sensitive to arousal and valence dimensions of core emotions. The decoded information from the signals can be hardly masked due to the fact that cortical bioelectrical activities are directly recorded on the scalp. Similar observations can be found when using heart rate variability, electrooculargrams, and thermal inferred imaging [7] techniques.

It is also worth noting that a contactless physiological measurement, functional inferred imaging (fIRI), was applied in the domain of affective computing [7,8]. The advantage of the fIRI is to measure the facial cutaneous temperature in a remote and noninvasive manner. The fIRI shows its capability to indicate discrete emotions by reflecting the activity of the peripheral nervous system. In a recent work [8], it was implemented for evaluating emotional variations of children when interacting with a commercial educational robot system. In their work, a total of 31 participants were involved and perform oral interactions with the robot with speech recognition functionality. The computational psychophysiology module was used to map the fIRI based thermal data to arousal/valence dimensions. An overall accuracy of 71% was achieved by the multilayer perceptron classifier on negative, neutral and positive affective states. Variations in physiological signals are particularly convincing and reflect true emotional states in an objective way [2]. Among neurophysiological responses, electroencephalography (EEG) scans transitions in spontaneous activities in multiple cortical regions, which originate human emotional processes. The EEG has high sensitivity and specificity for distinguishing different emotional feedback [3,4]. In this study, we access raw EEG signals from three public databases (i.e., DEAP, MAHNOB-HCI, and SEED) to validate our machine-learning based emotion recognition (ER) framework.

Although EEG signals show optimistic performance in classifying emotions, different users of ER systems will have different cortical responses and mood swings to an identical emotional stimulus [9]. Thus, a main issue confronted in designing the ER system is the individual difference of the EEG response. However, an efficient ER system should be designed in a cross-subject paradigm. That is, the machine learning model should be trained by the EEG data recorded from a group of subjects (i.e., users of the ER systems) and tested on a novel subject. The advantage of such system lies in unnecessity of acquiring huge amount of data from a single individual.

It is notable that previous studies have shown that the accuracy of the cross-subject ER system is lower than that of the subject-specific version. Kim and André show that the best accuracy of the bimodal emotion recognition on all three subjects’ data was 55%, which is far below the accuracy (92.00%) when the training and testing data were drawn from the same subject [10]. Zhu et al. extracted differential entropy features of the EEG as the input of a linear support vector machine (SVM), where the accuracy of the subject-specific emotion recognition (90.97%) is much higher than that of the cross-subject condition (64.82%) [11]. It implied a classifier cannot effectively transfer knowledge pertaining to EEG data distribution among multiple users [12]. The individual difference leads to a difficulty for the generality and applicability of the extracted and selected EEG features.

To tackle this obstacle, we focus on designing the feature selection and fusion approach for transferring knowledge among multiple users’ EEG in the cross-subject ER system. Transfer learning uses prior knowledge and concepts in the source domain to apply to a target domain by adjusting the machine model to match the novel data distribution [13]. Transfer learning is widely used in image classification [14], fault diagnosis [15], data mining and knowledge discovery systems [16]. In particular, it facilitates a cross-subject ER system to predict correct emotional labels with insufficient EEG instances from a single subject since all user’s data can be exploited to train a generalizable model. To this end, we incorporate geodesic flow kernel (GFK) [17] to achieve unsupervised domain adaptation by sampling points in the estimated manifold [18].

In this study, we propose a cross-subject ER framework based on a novel EEG feature selection method termed as feature fusion and dynamical feature selection (MF-DFS). In the MF-DFS model, neighborhood component analysis (NCA) is first applied to reduce the dimensionality of the EEG features. Then, the GFK is used to map the fused EEG features to the Glassmann manifold space. Specifically, we propose a dynamical feature selection method (DFS) combined with the random forest (RF) to determine the most relevant features and improve the generalization capacity of the ER model.

This paper is structured as follows. Section 2 briefly reviews related works on methods of the EEG-based emotion recognition. In Section 3, we describe the EEG databases and approaches of feature selection and machine learning based classification. Comparison of the emotion classification performance is shown in Section 4. In Section 5, we discuss the main findings and point out the limitations of the present study. Finally, Section 6 concludes the study and lists our future work.

## 2. Related Works

We employ the DEAP, MAHNOB-HCI and SEED databases to validate the proposed ER framework. The DEAP was built by Koelstra et al. [19] and is used to study human emotion variations based on the multi-modal physiological data. In previous studies, the recognition accuracy of the valence and arousal dimensions on the DEAP can be achieved at 70.00% [20,21]. When deep neural networks are employed, the accuracies of arousal and valence are 61.25% and 62.50%, respectively. For convolutional neural networks and the RF, the accuracies are 88.49%/87.44% and 59.22%/55.70% for the arousal/valence dimension [22,23,24]. Among these studies, most of the classifiers are designed with subject-dependent paradigm, where training and testing data were drawn from the same subject. Under this context, bispectral analysis with the support vector machine (SVM) was developed and the accuracy of 72.50%/73.30% for arousal/valence was achieved [25]. Plataniotis et al. [26] adopted two types of semi-supervised deep learning approaches, stacked denoising autoencoder and deep belief networks, as feature extractors. The accuracies of valence and arousal dimensions are 88.33% and 88.59%, respectively.

The MAHNOB-HCI database was collected by Soleymani et al., where the EEG and physiological signals from the peripheral nervous system were available to indicate affective states of subjects [27]. Yan et al. proposed a modified common spatial pattern extractor and combined it with channel selection method, which achieved an average accuracy of the MAHNOB-HCI at 94.13% [28]. In our previous work, a locally robust feature selection method was proposed to find an EEG feature subset with local generalization ability among a group of subjects [29]. For two emotion classes under cross-subject paradigm, average classification accuracies on arousal and valence dimensions of the MAHNOB-HCI database are 67.00% and 70.00%, respectively. Tan et al. used a short-term ER framework based on a spiking neural network with spatio-temporal EEG patterns [30]. They segmented EEG signals and extracted their short-term changes and to avoid handcrafted feature engineering. The average accuracies of valence and arousal dimensions of the MAHNOB-HCI database are 72.12% and 79.39%, respectively.

The SEED database was built by the BCMI laboratory. Three (positive, negative and neutral) target emotions for each physiological data clip were induced when the subject was watching movie clips. Lu and Zheng built an EEG-based ER system by training deep belief networks (DBN). It is reported that the average recognition accuracy (86.65%) of four selected channels is higher than that (86.08%) of the 62 EEG channels [31]. Wang et al. proposed electrode-frequency distribution maps with short-time Fourier transform for the EEG feature extraction and applied it on the SEED database [32]. Residual block based deep convolutional neural network is used as the base classifier and the accuracy is 90.59%, which is 4.51% higher than the baseline [31]. Lu et al. developed a cross-subject ER system with the dynamic entropy model learning framework [33]. The average recognition accuracy of negative and positive emotions in the SEED database was 85.11%.

In recent works, machine learning approaches are also validated by using other EEG databases. Katsigiannis et al. has built the DREAMER database [34] which possessed multi-modality data from the EEG and electrocardiogram (ECG). Based on the support vector machine binary classifier. Accuracy for valence dimension reached 62.49% with the EEG modality, while the fusion of the EEG and ECG features provided the highest accuracy of the arousal dimension (62.32%). Baldo et al. [35] proposed a model for predicting consumer’s affective states on the novel products based on the EEG signals. They recorded EEG data of 40 participants while viewing the different shoes on the computer screen. Consumer’s preference, i.e., like or dislike, on each pair of the shoes with different fashions can be classified. Murugappan et al. [36] applied the k-nearest neighbor and probabilistic neural network to recognize emotional states of 12 participants towards different brand advertisement videos. By extracting power spectral density, spectral energy and centroid features of the EEG, the accuracy of 96.62% was achieved. Abadi’s lab present DECAF [37], a multimodal database for decoding user physiological responses to affective multimedia content. The brain activity has been scanned by magnetoencephalogram sensors. They used a linear support vector machine and achieved a mean accuracy of 57.9% and 51.25% over 30 participants using leave-one-subject-out cross-validation on arousal and valence dimensions, respectively.

In general, the design of the ER system can be categorized into two schemes, namely subject-specific and cross-subject. Although the accuracy of the last case is usually lower than that of the first two cases, there is no doubt that the cross-subject ER system requires less amount of the EEG instances from a specific individual. Previous studies indicate the differences of neuro-physiological responses between individuals brought difficulties to the cross-subject ER task compared to the subject-specific condition. To this end, we focused on designing the cross-subject EEG feature selection method and emotion classifiers and validated it on previously mentioned databases.

By briefly reviewing recent reported works, we notice that the individual difference of the EEG distribution significantly impairs the generalization capability of machine learning classifiers [26,27,28]. The reason behind is that the psychophysiological process induced by specific affective stimuli varies among different people [20,21,22,23]. A promising solution is to extract or select stable EEG features that are invariant across individuals [17,18,19,24]. In [17], the variational mode decomposition was used to discover these invariant spatial EEG features from raw signals. In [24], a locally robust feature selection method was used to quantify the consistency of the feature distribution of the same affective state among all BCI users. Encouraged by these works, in the present study we first apply the neighborhood component analysis and geodesic flow kernel to map EEG features from source and target domains to a Grassmann manifold space. Under this context, the source and target domains are built from different people. Therefore, consistency feature representations can be learned based on domain adaptation and knowledge transferring. We also develop a dynamical feature selection module to adaptively locate sensitive EEG features to emotional variations. The selected features can be properly adjusted when the training EEG data of a specific subject with abnormal feature distribution are employed.

## 3. Materials and Methods

**Figure 1:**
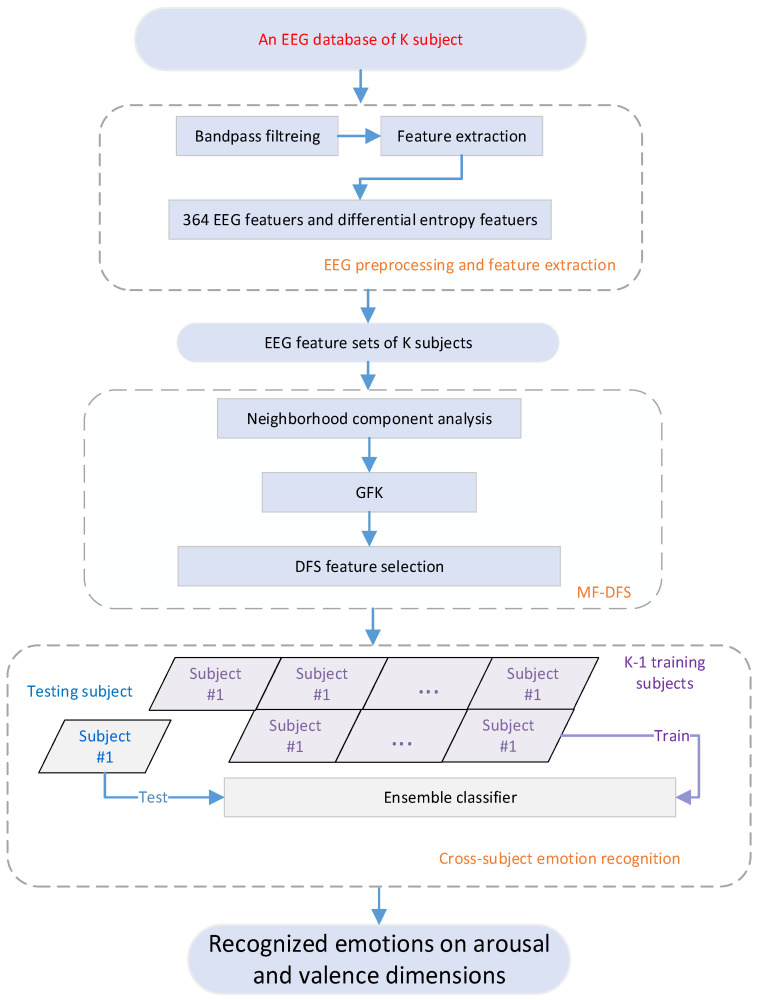
Emotion classification framework based on manifold feature fusion and dynamical feature selection method (MF-DFS).

In this section, the employed EEG databases were first introduced. Then, we provide procedures for EEG data preprocessing and assignment of the emotion labels. The steps of feature extraction are also described in detail. Finally, the mathematical method of the feature fusion and dynamical feature selection method is described. Two manifold learning techniques (the NCA and GFK) for feature fusion are reviewed. The detailed steps of the proposed DFS feature selection method is described. The framework of the proposed ER system is illustrated in Figure 1.

### 3.1. Database Descriptions

The DEAP dataset collected EEG and peripheral physiological signals from 32 channels. When the EEG signals were recorded, 40 selected music videos (1 min each) were viewed by 32 volunteers (16 males). The participants were asked to assess a self-assessment manikin questionnaire after watching each video. At the end of a trial, arousal, valence, dominance, liking and familiarity scales were rated by the volunteers within a range of 1–9. All the collected EEG signals are downsampled to 128 Hz. In this study, only the EEG signals are used for building the ER system.

The MAHNOB-HCI, a dataset of 30 volunteers, recorded physiological signals of electrocardiogram, EEG (with 32 channels), respiratory amplitude and skin temperature.

The EEG modality is used in building the ER system. Since the EEG data of six subjects are incomplete, only 24 participants’ data are available. Each participant rated arousal, valence, dominance, and predictability scales from 1 to 9 for 20 selective musical videos. The recorded EEG is downsampled to 128 Hz. We extract a 60-s EEG segment from each trial for further analysis. The first 5 s signal and the signal after 65 s of each trial are removed.

For the SEED database, 15 subjects participated in three trials of data acquisition experiments with an interval of 1 week between two consecutive trials. Each subject watched fifteen selected clips of Chinese films to stimulate target emotions on the valence scale, i.e., positive, neutral and negative. Each clip lasted approximately 4 min. The EEG data were simultaneously recorded with 62 channels and 32 channels are selected for further analysis. The selected 32 channels are as same as those used in the DEAP and the MAHNOB-HCI. The raw EEG signal was recorded at a frequency of 1000 Hz and then downsampled to 200 Hz.

### 3.2. EEG Data Preprocessing

In the process of the EEG acquisition, the signals can be interfered by artifacts induced by the ocular, muscular, and movement noise. Low and high pass filters are selectively employed to remove muscular noise or ocular disturbance. Compared to the SEED and DEAP databases, the EEG in the MAHNOB-HCI is required to be referenced. Since the movement and respiration artifacts are observed in the MAHNOB-HCI, a highpass filter with the cutoff frequency of 3 Hz is applied. For the DEAP, a bandpass filter with the cutoff frequencies of 4 and 45 Hz is implemented. For the SEED, a highpass filter (3 Hz) is first used to remove the motion and respiratory artifacts. Then, a lowpass filter (45 Hz) is used to eliminate high frequency noise. Table 1 shows the detailed implementations of the filter settings of the three databases. To generate sufficient training instances of the ER classifier, we divide a trial EEG data into four nonoverlapped segments for all three databases. In total, 5120 (160 for each subject), 1920 (80 for each subject) and 2700 (180 for each subject) EEG segments are elicited from the DEAP, MAHNOB-HCI and SEED databases, respectively.

Since the supervised machine learning models are used for building the ER system, we assign target emotion classes to each EEG segment as follows. The emotion class for each trial is fixed. For the DEAP and MAHNOB-HCI databases, three target classes according to the subjective ratings on arousal and valence dimensions are assigned for each trial. A rating value higher than 5.5 indicates the high class, the value between 5.5 and 3.5 indicates the neutral class, and the value lower than 3.5 indicates a low class. Hence, for an affective dimension of arousal or valence, three target emotions are defined and assigned to each trial of the EEG data. For the SEED database, all trials have been categorized into three emotional classes (positive, neutral, negative).

### 3.3. Feature Extraction

The preprocessed EEG signals of each segment are then transformed to a feature vector aiming at sensitively indicating emotion variations. In this study, we incorporate 364 classical EEG features (CL) [38] and 128 differential entropy (DE) features [39] from the signals of 32 EEG channels.

#### 3.3.1. Classical EEG Features

For all three EEG databases, 364 CL (204 frequency-domain features and 160 time-domain features) are extracted and shown in Table 2. Considering the asymmetry of the left and right hemispheres, power differences between left and right hemispheres of scalps in four frequency bands (theta: 4–8 Hz, alpha: 8–14 Hz, beta: 14–31 Hz, gamma: 31–45 Hz) were extracted. The power features of the same four bands of 32 channels were calculated by the fast Fourier transform. Power ratios of specific channels and frequency bands were also calculated. At the same time, we extracted five sets of time-domain features as shown in Table 2.

#### 3.3.2. Differential Entropies

The DE is an extension of the Shannon entropy and has been widely applied for building the EEG-based ER systems [40]. In this study, we compute the DE for each classical band defined as follows,

(1) $$h(X)=-\int_{X}f(x)\log\left(f(x)\right)dx.$$

According to Equation (1), f(x) is the probability density function of the EEG time series X in a specific frequency band after bandpass filtering. According to the Kolmogorov–Smirnov statistic [41], the filtered EEG signals were the time series of Gaussian distribution $N\left(\mu ,\sigma^{{}^{2}}\right)$. Therefore, Equation (1) can be approximately computed based on the variance of the filtered.

(2) $$\begin{array}{l} h\left(X\right)=-\int_{-\infty }^{\infty }\frac{1}{\sqrt{2\pi \sigma^{2}}}e^{{\left(x-\mu \right)}^{2}/(2\sigma^{2})}\pi \log\left(\frac{1}{\sqrt{2\pi \sigma^{2}}}e^{{\left(x-\mu \right)}^{2}/(2\sigma^{2})}\right)dx\\ \;=\frac{1}{2}\log\left(2\pi e\sigma^{{}^{2}}\right). \end{array}$$

### 3.4. Manifold Feature Fusion and Dynamical Feature Selection

In this section, the proposed MF-DFS framework for fusing and selecting EEG features for building the cross-subject ER system is described in detail. We first briefly review the NCA and GFK feature fusion methods. Then, the details of the proposed DFS feature selection method are shown.

#### 3.4.1. Neighborhood Component Analysis

The NCA [42] is a non-parametric method for selecting features with the goal of maximizing prediction accuracy of the classifier. It has a capability to learn the Mahalanobis distance between training instances and linearly transform them to a subspace such that the average cross validation classification accuracy is maximized. The motivation of applying the NCA method lies in two aspects. First, the NCA employs a stochastic 1-nearest neighbor (1-NN) classifier to examine whether the predicted class is consistent with the target class of the EEG features. Compared to unsupervised principal component analysis, the NCA adopts supervised learning principle that could exploit label information to increase interclass distinguishability. Moreover, the comparison between the predicted and target classes is based on leave-one-out cross validation. Compared to artificial neural network with empirical risk minimization, it better controls the overfitting when performing metric learning of the distance weight. It should be noted that the functionality of the 1-NN classifier is to evaluate the distance of each two instances belonging to the same class. It facilitates that the low dimensional features are embedded with large inter-class margin, which is different from the k-NN classifier directly used for classification. In this study, the fold for the cross validation is 15.

A multi-class training set T of n samples can be defined as $T=\left\{\left(X_{i},y_{i}\right),i=1,2,\ldots ,n\right\}$, where X are d-dimensional feature vectors, $y_{i}\in \left\{1,2,\ldots ,C\right\}$ are the class labels, and C is the number of class. The aim is to learn a classifier to generate prediction f(X). The prediction should be close to the true label y of X. To select the optimal feature subset, we define $D_{w}$ as the weighted distance between samples $X_{i}$ and $X_{j}$. In this scheme, a reference point is randomly chosen to be the nearest neighbor of the new point X. The probability that a point $X_{j}$ is picked from T as the reference point for X increases if $X_{j}$ is closer to X as measured by the distance function $D_{w}$,

(3) $$D_{w}\left(X_{i},X_{j}\right)=\sum_{l=1}^{d}w_{r}^{2}\left|X_{ir}-X_{jr}\right|,$$

where $w_{r}$ is the feature weight.

The leave-one-subject-out paradigm is then applied to evaluate the classifier’s performance. That is, the predicted label of $X^{\left(i\right)}$ is generated by the classifier trained by the dataset $T^{\left(-i\right)}$ that denotes excluding the subset of the training instances $\left(X^{\left(i\right)},y^{\left(i\right)}\right)$ from the training set T. The probability that a point $X_{j}$ is picked as the reference point for $X_{i}$ is,

(4) $$p_{ij}=\{\begin{array}{c} \frac{k\left(D_{w}\left(X_{i},X_{j}\right)\right)}{\sum_{j=1}^{n}k\left(D_{w}\left(X_{i},X_{j}\right)\right)},\;\mathrm{if}\;i\neq j\\ 0,\;\;\;\;\;\mathrm{if}\;i=j \end{array}.$$

In Equation (4), kernel function k(z)=exp(−z/σ) achieves a large value when $D_{w}\left(X_{i},X_{j}\right)$ decreases. The kernel width σ influences the probability of a training sample being selected as the reference. The $p_{i}$ is the probability that the classifier correctly classifies the data point using the training dataset T.

(5) $$p_{i}=\sum_{j=1,j\neq i}^{n}p_{ij}y_{ij}.$$

In Equation (5), $y_{ij}$ can be elicited as.

(6) $$y_{ij}=I(y_{i}=y_{j})=\{\begin{array}{c} 1\;\mathrm{if}\;y_{i}=y_{j}\\ 0\;\mathrm{otherwise} \end{array}.$$

Thus, the average probability of correct classification is derived as,

(7) $$F=\frac{1}{n}\sum_{i=1}^{n}p_{i}.$$

The goal of the NCA is to maximize F to improve the classification accuracy. To reduce the overfitting, a regularization parameter λ>0 is induced to balance the F and the summation of the weights [43]. In this study, the kernel width σ is simply selected as 1. The objective function in Equation (7) can be generalized as,

(8) $$F(w)=\frac{1}{n}\sum_{i=1}^{n}p_{i}-\lambda \sum_{r=1}^{p}w_{r}^{2}.$$

To find the proper value of and determine the dimension of the selected features, the leave-one-subject-out is applied again as follows:

1. Partition the EEG feature data into K subsets and each subset contains the EEG data of a subject;
2. Perform K-fold leave-one-subject-out validation;
3. For each fold, train a NCA model on K-1 subsets and validated the trained model on the remaining subset;
4. Return the value of the classification loss defined as the mean square error for the current fold;
5. Repeat steps (2)–(4) to find the lowest loss corresponding to optimal the value of λ;
6. Perform NCA feature selection according to the optimal λ.

#### 3.4.2. Geodesic Flow Kernel

**Figure 2:**
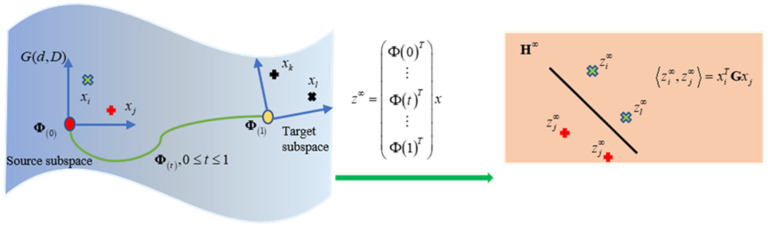
Basic idea of the GFK for domain adaptation.

The geodesic is defined as the shortest local distance between two points in the feature space. To find a geodesic, the source and target domains are mapped to a Grassmann manifold space [17] as shown in Figure 2. Given two points projected on the Glassmann manifold, the kernel method is used to select all the geodesic points from the source to target domains with seamless migration. There are three steps to build a GFK model.

(1) Obtain the optimal dimension of the subspaces.

The GFK adopts the subspace disagreement measure (SDM) to find the intrinsic dimension of the subspace. The SDM D(d) is defined as,

(9) $$D(d)=0.5(\sin\alpha_{d}+\sin\beta_{d}).$$

Given two datasets S and T, the principal component analysis (PCA) is applied and obtain subspaces $P_{S}$ and $P_{T}$, respectively. Then, a dataset S+T is created by combining S and T, the PCA is applied again to derive the subspace $P_{S+T}$. The term $\alpha_{d}$ (or $\beta_{d}$) represents the *d*th principle angle between $P_{S}$ and $P_{S+T}$ (or $P_{T}$ and $P_{S+T}$) [44]. The only hyper-parameter needs to be tuned is the dimensionality of the subspaces d. The value of the d is minimized with the constraint of D(d)=1. The constraint ensures the basis of the $P_{S}$ or $P_{T}$ is orthogonal to that of the $P_{S+T}$.

(10) $$d^{*}=\min\left\{d|D\left(d\right)=1\right\}.$$

A larger value of d is preferred to contain more information from the fused features.

(2) Build geodesic flow.

After implementing the PCA, all *d*-dimensional subspaces are embedded into manifold H. The terms S and T represent the subspaces of source and target domain, respectively. Then, H can be regarded as the set of all *d*-dimensional subspaces. Every possible subspace in *d* dimensions can be considered as a point on H. Thus, a geodesic between two points can form a path between two subspaces.

Suppose that the subspaces of the source and target domains are projected by a geodesic mapping function Φ, and assume that they are in two poles of 0 and 1 in the manifold space, there exists $\Phi_{0}=P_{s}$ and $\Phi_{1}=P_{T}$. Let $R_{S}\in R^{D\times (D-d)}$ denote the orthogonal complement to $P_{s}$, and $R_{S}^{T}P_{S}$. For a point t mapped within the interval of [0, 1], the corresponding mapping function is defined as $\Phi_{t}$. This function can be computed as [44,45],

(11) $$\Phi_{t}=P_{S}U_{1}\Gamma_{t}-R_{S}U_{2}\Sigma_{t}.$$

In the equation, $U_{1}$ and Γ are elicited by the $P_{S}^{T}P_{T}=U_{1}\Gamma V^{T}$ according to the singular value decomposition (SVD) while $U_{2}$ and Σ are computed via $R_{S}^{T}P_{T}=-U_{2}\Sigma V^{T}$. It is noted that the diagonal element of Γ can be represented as $\cos\theta_{i}$ while that of Σ is $\sin\theta_{i}$, where $\theta_{i}$ denotes the principle angle between $P_{S}$ and $P_{T}$ with $0\leq \theta_{1}\leq \theta_{2}\leq \ldots \leq \theta_{d}\leq \pi /2$.

(3) Calculate the geodesic flow kernel.

For two vectors $x_{i}$ and $x_{j}$, their projections on $\Phi_{t}$ can be represented as infinite-dimensional vector: $z_{i}^{\infty }$ and $z_{j}^{\infty }$. The inner product of $z_{i}^{\infty }$ and $z_{j}^{\infty }$ defines a geodesic stream kernel,

(12) $$<z_{i}^{\infty },z_{j}^{\infty }>=\int_{0}^{1}{\left(\Phi {\left(t\right)}^{T}x_{i}\right)}^{T}(\Phi {\left(t\right)}^{T}x_{j})dt=x_{i}^{T}Gx_{j}.$$

In the equation, $G\in R^{D\times D}$ can be calculated by the following closed-form SVD [6],

(13) $$G=\left[\begin{array}{cc} P_{S}U_{1} & R_{S}U_{2} \end{array}\right]\left[\begin{array}{cc} \Lambda_{1} & \Lambda_{2}\\ \Lambda_{2} & \Lambda_{3} \end{array}\right]\left[\begin{array}{cc} U_{1}^{T} & P_{S}^{T}\\ U_{2}^{T} & R_{S}^{T} \end{array}\right].$$

Thus, through the GFK mapping, the source domain features are transformed into a Grassmann manifold with Equation (13). The mapping matrix $\sqrt{G}$ can be computed based on the SVD decomposition.

#### 3.4.3. Dynamical Feature Selection and Performance Evaluation of the MF-DFS

The aim of the feature selection to find a relevant feature subset with lower dimensionality and less noise. In this work, we propose a novel DFS method to find the most informative EEG variables indicating variations of the emotions. The DFS is developed based on recursive feature elimination (RFE) approach. The RFE was proposed by Guyon et al. and originally used for the gene selection task [46]. The RFE is a wrapper-based feature selection method and adopts a sequential backward elimination strategy.

The aim of the DFS is to reduce the differences between individuals and achieves a more consistent probability distribution across the source and target domain. It is noted that the DFS should be implemented with a predefined emotion classifier. It is used to generate the weight of fused features. The input feature matrix $X_{DFS}$ of the DFS is calculated as,

(14) $${X_{DFS}=\sqrt[{}]{G}X_{NCA}}^{T}.$$

In the equation, $X_{NCA}$ is elicited by the NCA based feature selection and $\sqrt[{}]{G}$ is derived based on Equation (13).

The procedure for implementing the DFS is summarized as follows.

1. Perform the leave-one-subject-out training and testing procedure;
2. Select a CL or DE feature set from a database with N subjects and compute the corresponding feature matrix $X_{NCA}$;
3. Define a testing set, where the EEG data are drawn from a specific subject;
4. A predefined emotion classifier is trained by the learning algorithm L based on the remaining N−1 subjects’ EEG data. The dimension of the EEG feature is defined as n;
5. Perform feature ranking according to the feature weights according to the trained classifier;
6. Remove the feature with the lowest weight and update the feature matrix;
7. Retrain the SVM classifier based on the current feature matrix and update the weight;
8. Repeat steps (5)–(7);
9. Generate a feature ranking according to the order of the feature removal. The first (or last) removed feature possesses the lowest (or highest) ranking;
10. Given the classifier, compute n classification accuracies. For instance, the 1st accuracy corresponds to that the optimal feature is adopted according the feature rankings to train the classifier, the 2nd accuracy indicates the optimal two features are adopted, and the *n*th accuracy indicates all features are used;
11. Determine the optimal feature combination corresponding to the highest accuracy elicited in step (10);
12. Repeat steps (3)–(11) for all testing subjects.

The generalization capability of the proposed MF-DFS model is validated by the leave-one-subject-out cross validation paradigm combined with specific machine learning classifiers. We take the DEAP database with the EEG recorded from 32 subjects as an example. The EEG features are first fused and selected by the MF-DFS model and then divided into 32 subsets. Each subset contains the EEG data with the reduced dimensionality from an individual. The machine learning classifier for classifying low and high arousal/valence classes is trained based on 31 subsets and validated on the remaining subset. Therefore, each subset can be validated once. After all subsets are validated for 32 rounds of such training and testing procedure, the average classification accuracy is computed as the performance of the MF-DFS method. For the HCI and SEED databases, 24 and 15 subsets are built according to the number of the individuals, respectively.

## 4. Results

### 4.1. NCA Model Selection

**Figure 3:**
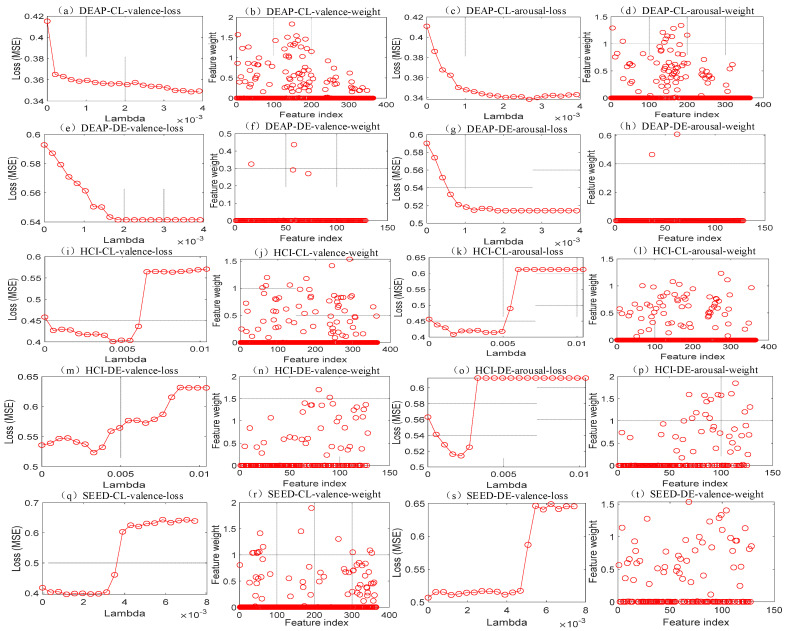
Model selection of the NCA. Subfigures (**a**,**b**) denote the loss values and the NCA weight for the CL features from the DEAP database of the valence dimension. Subfigures (**c**,**d**) denote the loss and NCA weight for the CL features from the DEAP of the arousal dimension. Subfigures (**e**,**f**) denote the loss and NCA weight for the DE features from the DEAP of the valence dimension. Subfigures (**g**,**h**) denote the loss and NCA weight for the DE features from the DEAP database of the arousal dimension. Subfigures (**i**,**j**) denote the loss and NCA weight for the CL features from the MAHNOB-HCI database of the valence dimension. Subfigures (**k**,**l**) denote the loss and NCA weight for the CL features from the MAHNOB-HCI of the arousal dimension. Subfigures (**m**,**n**) denote the loss and NCA weight for the DE features from the MAHNOB-HCI of the valence dimension. Subfigures (**o**,**p**) denote the loss and NCA weight for the DE features from the MAHNOB-HCI of the arousal dimension. Subfigures (**q**,**r**) denote the loss and NCA weight for the CL features from the SEED database of the valence dimension. Subfigures (**s**,**t**) denote the loss and NCA weight for the DE features from the SEED of the valence dimension.

To achieve the optimal performance of the proposed MF-DFS method, the hyper-parameter λ of the NCA is carefully determined based on the leave-one-subject-out cross validation. For each feature set (CL or DE), the average loss (mean square error, MSE) of all folds are computed. Note that the number of features in the NCA across different features sets varies within an interval of 27–38. The optimal λ with the smallest MSE is applied in the NCA model. The variations of the MSE vs. corresponding values of λ for all feature sets and emotional dimensions are shown in Figure 3. Taken Figure 3a as an example, the best loss of 0.34 is achieved corresponding to the λ value of 0.0037. In Figure 3b, the corresponding weights of the DE feature set of the DEAP database are shown. We can observe most weight values are zeros, which identify an irrelevant feature subset. Thus, the number of the selected features and the optimal value of λ can be simultaneously determined for each feature set of all databases according to Figure 3. It should be noted that we also adopt a threshold to control the number of the selected features when most of the weights are zeros or non-zeros.

The feature importance to variations of the emotion can be interpreted by the NCA weight shown in Figure 3. By averaging weight values of all EEG channels and databases, beta (0.1694) and gamma (0.1622) bands possess higher importance than that from theta (0.0502) and alpha (0.0699) bands with respect to the valence dimension. For arousal variations, similar observations are shown with the averaged weight of 0.0175, 0.0447, 0.1516 and 0.1546 for the theta, beta, beta and gamma bands, respectively. By sorting the weight in a descending order, the most important channels to the valence for the beta band are the Pz and O1. It implies an increased cortical response in parietal and occipital regions. The most important channels to the valence for the gamma band are Fp1 and F7, which shows an increased cortical activity in the left frontal region. For the arousal dimension, the most important features in the beta band are T7 and Fp2 while those in the gamma band are Fp1 and F8. In conclusion, the features that are sensitive to emotion variations are identified from beta and gamma power in central parietal, left occipital, frontal and left temporal regions of the scalp.

### 4.2. Feature Selection Performance with Different Classifiers

To further validate the performance of the MF-DFS based ER systems, five classifiers, random forest (RF), adaptive boosting (AdaBoost), gradient boosting decision tree (GBDT), extreme gradient boosting (XGBoost) and decision tree (DT) are applied. All hyper-parameters of the classifiers have been carefully selected and listed in Table 3. The selected hyper-parameters are fixed under all cases of the experiments.

In Table 4, Table 5 and Table 6, we compare the accuracy of the CL and DE feature sets under five feature selection methods, i.e., Chi-squared-based feature selection (CSBS), mutual information-based feature selection (MI), ridge regression-based feature selection (RR), extremely random forest (ERF), and the proposed DFS. All accuracies are computed based on the inter-subject manner based on the leave-one-subject-out paradigm. Training data of all classifiers are processed based on the NCA and GFK. Therefore, the last column of each table shows the results of the MF-DFS. In total, there are 25 combinations of different feature selection methods and classifiers. For the DEAP and MAHNOB-HCI databases, the arousal and valence dimensions of the emotions are recognized. For the SEED database, only the valence dimension is evaluated since the arousal targets are unavailable.

From the tables, it is shown the classification accuracies of the DFS combined with all five classifiers are significantly higher than the other four feature selection methods for all three databases. Moreover, it can be found that the DFS combined with the RF possesses the highest average classification accuracy (0.4236). In addition, in the comparison of the two feature sets in three databases, the CL feature has a higher average classification accuracy than the DE feature set for both arousal and valence dimensions. The average accuracy of DEAP database CL feature is 0.4470, and that of DE feature is 0.4381. For the MAHNOB-HCI database, the average accuracies for two feature sets are 0.4178 and 0.4062. For the SEED database, the average accuracies for two feature sets are 0.3465 and 0.3450. It implies the emotions in the DEAP database possess higher distinguishability.

In Table 4, Table 5 and Table 6, five classifiers were validated based on different feature selection and fusion techniques. For the DEAP database, the AdaBoost combined with the MF-DFS achieves the optimal performance on both of classical and differential entropy features for arousal and valence dimensions. The improvement is approximately 0.2–1.5% averaged for all cases against other combinations in Table 4. For the MAHNOB-HCI database, the RF combined with the MF-DFS is superior to other cases with the accuracy improvement of 1–3.7%. For the SEED database, the improvement of the DT with 0.6–1.8% is observed. Overall, the RF model outperforms the other classifiers averaged by all three tables. The potential reason lies in two aspects. The RF employs a group of member classifiers to build a classification committee by majority voting. A hyper-parameter is required to be tuned, i.e., number of the member classifiers. By using the proper amount of member classifiers, the fitting capability can be superior to that of the DT with only a single classification model. Moreover, the RF employs random sampling simultaneously on training instances and input EEG features, which is different from the AdaBoost and classical ensemble method only adopting instance sampling. The training subset can be built based on the bootstrap approach using a lower feature dimension. It thus potentially reduces the overfitting of the member decision trees.

### 4.3. Statistical Test of Feature Selection Performance

**Figure 4:**
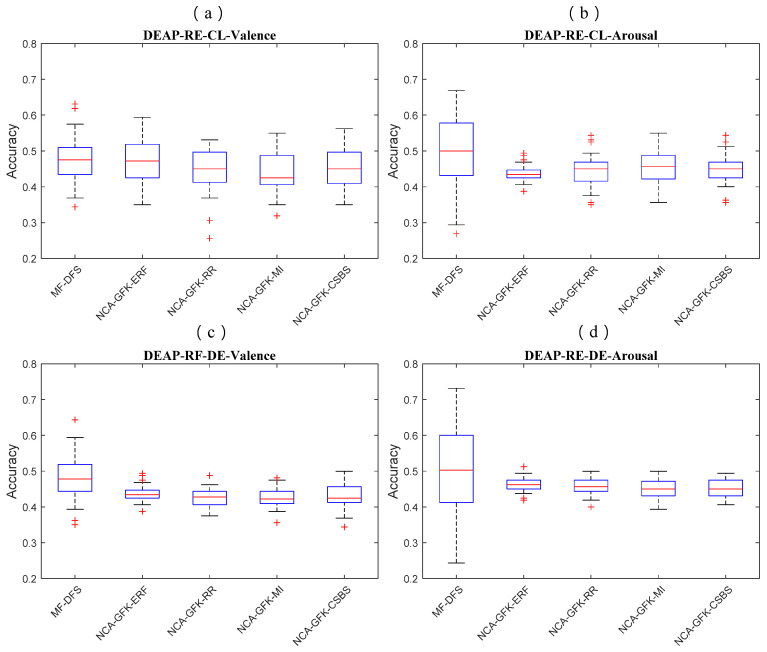
Boxplots of the classification accuracy based on the RF classifier combined with five feature selection methods of the DEAP database. The accuracies of CL features for the valence and arousal dimensions are shown in (**a**,**b**), respectively. The accuracies of DE features for the valence and arousal dimensions are shown in (**c**,**d**), respectively.

**Figure 5:**
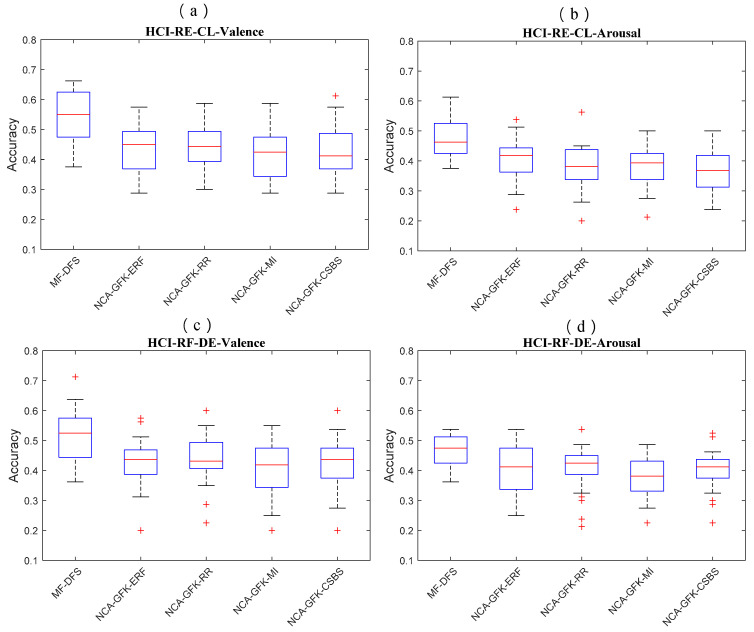
Boxplots of the classification accuracy based on the RF classifier combined with five feature selection methods of the MAHNOB-HCI database. The accuracies of CL features for the valence and arousal dimensions are shown in (**a**,**b**), respectively. The accuracies of DE features for the valence and arousal dimensions are shown in (**c**,**d**), respectively.

**Figure 6:**
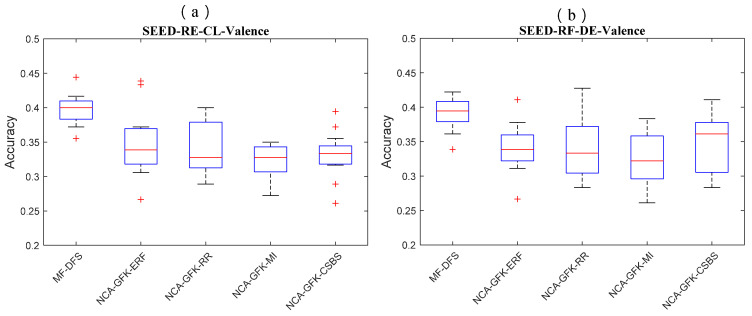
Boxplots of the classification accuracy based on the RF classifier combined with five feature selection methods of the SEED database. The accuracies of CL features for the valence dimension are shown in (**a**). The accuracies of DE feature for the valence dimension are shown in (**b**).

In Figure 4, Figure 5 and Figure 6, we show of the RF classifier combined with five feature selection methods of the three databases. In Figure 4, the MF-DFS achieves the optimal accuracy on both CL and DE feature sets. For the CL feature set, the MF-DFS combined with the RF classifier achieves a recognition accuracy of 0.48 and 0.5 for valence and arousal dimension, respectively. For the DE feature set, the corresponding accuracies of the valence and arousal are 0.48 and 0.5, respectively. In Figure 5, for the MAHNOB-HCI database, the accuracy of the CL feature set is 0.54\0.48 (valence\arousal) and that of the DE feature set is 0.51\0.47 (valence\arousal). In Figure 6 for the SEED database, the valence accuracy of the CL and DE feature sets are 0.40 and 0.40, respectively. It indicates the performance of the proposed MF-DFS method achieves higher median than other feature selection methods. It should be noted the accuracies of the MF-DFS share a larger variance for the arousal dimension of the DEAP database. It implies the cross-subject classification fails on specific individuals.

**Figure 7:**
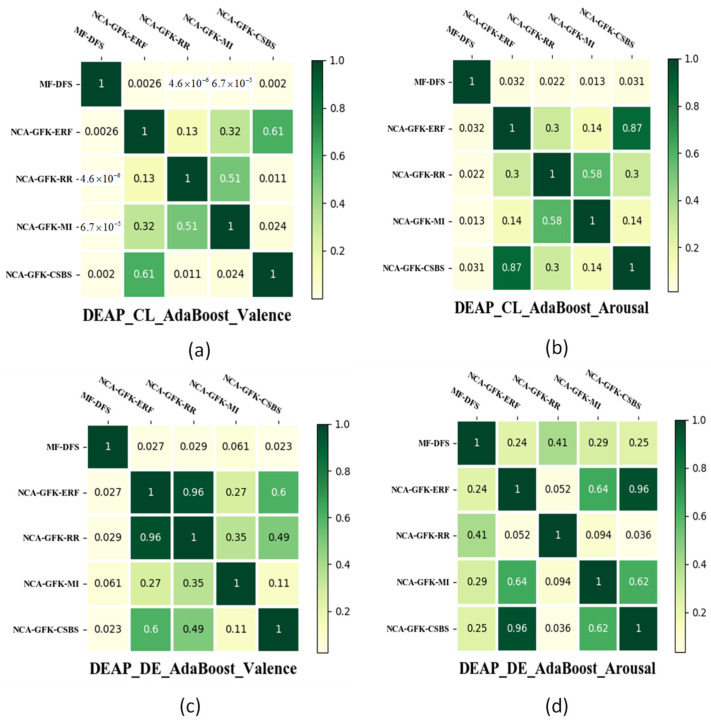
Heat map of the *p*-values of the paired *t*-test between different feature selection methods for the DEAP database. The *p*-values of CL features for the valence and arousal dimensions are shown in (**a**,**b**), respectively. The *p*-values of DE features for the valence and arousal dimensions are shown in (**c**,**d**), respectively.

**Figure 8:**
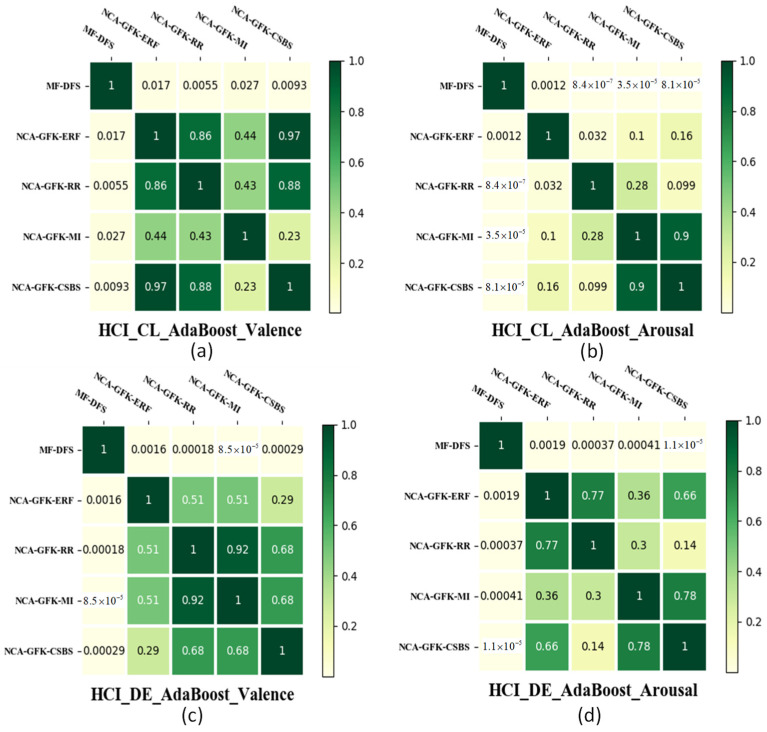
Heat map of the *p*-values of the paired *t*-test between different feature selection methods for the MAHNOB-HCI database. The *p*-values of CL features for the valence and arousal dimensions are shown in (**a**,**b**), respectively. The *p*-values of DE features for the valence and arousal dimensions are shown in (**c**,**d**), respectively.

**Figure 9:**
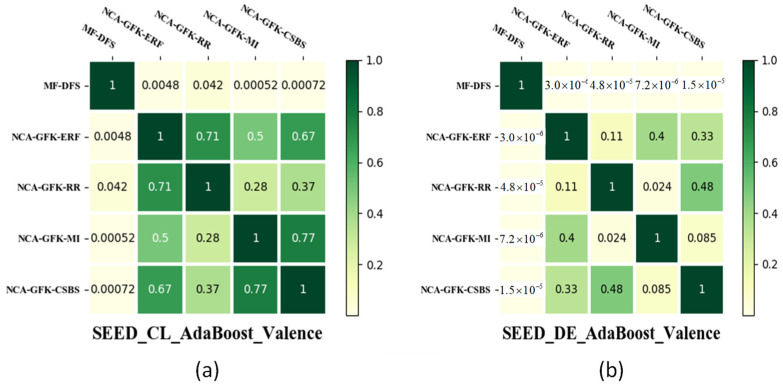
Heat map of the *p*-values of the paired *t*-test between different feature selection methods for the SEED database. The *p*-values of CL feature for the valence dimension are shown in (**a**). The *p*-values of DE features for the valence dimension are shown in (**b**).

In Figure 7, the results of the paired t-test are shown to compare whether the improvement between the MF-DFS and other feature selection methods is significant or not. To achieve a fair comparison, the ER classifier is fixed as the AdaBoost. In Figure 7, it can be observed that the MF-DFS significantly outperforms the remaining four feature selection methods with *p* < 0.05 for the valence dimension and the CL feature set of the arousal dimension. The difference between DFS and the other four feature selection methods is insignificant with the DE feature set for the arousal dimension. In Figure 8 and Figure 9, the significant improvement of the MF-DFS for the MAHNOB-HCI and SEED databases are observed across all cases and feature sets.

### 4.4. Performance Comparison between the MF-DFS and Original EEG Features

In this section, we compare the MF-DFS with the baseline state, where the extracted classical features and differential entropy are directly fed to the classifiers. The derived cross-subject emotion classification accuracy is presented in Table 7, Table 8 and Table 9. For the DEAP database, the performance of the average classification accuracies on valence and arousal dimensions of all adopted five classifiers of the two feature sets are all improved by 5.41%. For the MAHNOB-HCI database, the accuracy of classical features set is increased by 8.30% and that of differential entropy is increased by 8.24%. For the SEED database, the average classification accuracies of the classical and differential entropy feature sets are improved by 3.82% and 5.95%, respectively. The results show the competency of the MF-DFS model against the case using the original EEG features without proper feature selection.

## 5. Discussion

In this study, a three-class ER system based on the NCA-GFK feature fusion and the DFS feature selection has been proposed. Specifically, the ER system is developed with the cross-subject paradigm. Due to individual differences among the subjects in each database, such ER system is relatively difficult to share a satisfactory generalization capability. In practice, large amount of EEG data from a specific subject are difficult to acquire. Thus, efficient identification of the relevant EEG features between different individuals related to emotional variations is particularly critical.

The MF-DFS combines manifold feature fusion techniques and dynamical feature selection approach to achieve domain adaptation and knowledge transferring of the EEG statistics. It successfully reduces the dimension of the features and improves transferability across classical PSD and differential entropy feature sets of different individuals. In particular, the leave-one-subject-out accuracy of the proposed DFS significantly outperforms four competitive feature selection methods. The potential reason is the introduction of a dynamical feature filter aiming at adapting the personality of the EEG distribution of each user of the brain computer interface. The fairness of the comparison can be ensured since both NCA and GFK are leveraged to discover the proper manifold for all feature selection methods. By analyzing the cross-subject classification accuracy, the proposed MF-DFS has a capability to improve the accuracy of individual-independent emotion recognition in three different physiological databases.

The limitations of the proposed ER framework mainly manifest in the following two points. (1) The accuracy of the cross-subject emotion classification accuracy is still lower than 50% for three classification cases. It is still an obstacle for practical application for online implementation of the algorithms. (2) The essential of the DFS is a dynamic recursive process and it induces a high computational cost for finding the most relevant features.

## 6. Conclusions

In this study, we proposed an EEG feature selection method termed as the MF-DFS. It is specifically designed for cross-subject emotion recognition. The MF-DFS adopted the merits of local geometrical information-based feature selection (NCA), manifold estimation with domain adaptation (GFK) and dynamical feature selection to boost the performance of emotion classifiers. We validated the MF-DFS based on classical and differential entropy feature sets from three EEG database: the DEAP, MAHNOB-HCI and SEED. We observed the MF-DFS significantly outperforms classical feature selection methods on five machine learning classifiers. It partially demonstrated its generalization capability and transferability for inter-individual EEG feature selection. The future work will focus on the aspect of reducing the computational cost in its recursive procedure and further improve its usability for its practical application.

## Figures and Tables

**Table 1:** Filter implementations for the EEG in three databases.

Preprocessing Steps	Annotations	Applied Databases
Channel selection	32 channels: Fp1, AF3, F3, F7, FC5, FC1, C3, T7, CP5, CP1, P3, P7, PO3, O1, Oz, Pz, Fp2, AF4, Fz, F4, F8, FC6, FC2, Cz, C4, T8, CP6, CP2, P4, P8, PO4, and O2 according to the 10–20 system.	All
Downsampling	The downsampled frequency of the DEAP and HCI was 128 Hz and for the SEED it is 200 Hz.	All
Rereferencing	Subtract the average amplitude of all 32 channels.	MAHNOB-HCI
Bandpass-filtering	Five-order Butterworth filter with cutoff frequencies of 4 and 45 Hz.	All
Highpass-filtering	Seventh-order Butterworth filter with cutoff frequency of 3 Hz.	MAHNOB-HCI and SEED
Lowpass-filtering	Seventh-order Butterworth filter with cutoff frequency of 45 Hz.	SEED
Data segmentation	Data segmentation	All

**Table 2:** Descriptions of the extracted EEG features.

Feature Type	Feature Description	Feature Dimension
Classical features	Variation Zero Crossing Rate 1 Shannon Entropy Kurtosis Skewness	160
	6. Average Power Spectral Density (PSD) Average power of the frequency band in 4–8 Hz, 9–12 Hz, 13–30 Hz, and 31–45 Hz of each EEG channel, respectively.	128
	7. PSD Differences of the Four Bands Frontal scalp: $p_{Fp2}-p_{Fp1}$, $p_{AF4}-p_{AF3}$, $p_{F4}-p_{F3}$, $p_{F8}-p_{F7}$, $p_{FC6}-p_{FC5}$, $p_{FC2}-p_{FC1}$ Central scalp: $p_{C4}-p_{C3}$, $p_{T8}-p_{T7}$, $p_{CP6}-p_{CP5}$, $p_{CP2}-p_{CP1}$ Parietal scalp: $p_{P4}-p_{P3}$, $p_{P8}-p_{P7}$, $p_{PO4}-p_{PO3}$, $p_{O2}-p_{O1}$	56
	8. Power Ratio $p_{Fz}/(p_{AF3}+p_{AF4})$, $p_{Cz}/p_{Fz}$, $p_{Pz}/p_{Cz}$, $p_{Oz}/p_{Pz}$, $p_{AFz}(\theta )/p_{Pz}(\alpha )$, $p_{AFz}(\theta )/p_{Cz}(\alpha )$, $p_{Cz}(\theta )/p_{Pz}(\alpha )$, $p_{Cz}(\theta )/p_{Oz}(\alpha )$.	20
Differential entropy	The DE values of the frequency band in 4–8 Hz, 9–12 Hz, 13–30 Hz, and 31–45 Hz of each EEG channel.	128 ^1^

^1^ The subscription indicates the channel locations. The term *p* indicates the PSD value of four bands (theta, alpha, beta, and gamma), the marker “-” denotes a channel pair for computing the power difference, and the term *p*(*x*) denotes the power value on frequency band *x*. The marker “/” defines the ratio between two PSD values.

**Table 3:** Hyper-parameter settings for the ER classifiers.

Classifier	Hyper-Parameter Settings
RF	Number of the estimators = 50
AdaBoost	Number of the estimators = 50, maximum depth = 24
GBDT	Number of the estimators = 50, maximum depth = 16
XGBoost	Number of the estimators = 50, maximum depth = 22
DT	Maximum depth = 10, minimum samples in the leaf node = 12

**Table 4:** Classification performance comparison across different combinations of feature selection methods and machine learning classifiers on the DEAP database.

Performance	Classifier	Feature Selection Method				
		NCA-GFK-CSBS	NCA-GFK-MI	NCA-GFK-RR	NCA-GFK-ERF	MF-DFS
CL-Average valence	RF	0.4490 (5.60 × 10^−2^)	0.4445 (5.59 × 10^−2^)	0.4449 (6.26 × 10^−2^)	0.4678 (5.10 × 10^−2^)	**0.4791** (6.63 × 10^−2^)
	AdaBoost	0.4455 (4.74 × 10^−2^)	0.4283 (5.48 × 10^−2^)	0.4230 (6.12 × 10^−2^)	0.4400 (5.56 × 10^−2^)	**0.4959** (7.76 × 10^−2^)
	GBDT	0.4328 (5.05 × 10^−2^)	0.4357 (4.71 × 10^−2^)	0.4254 (6.83 × 10^−2^)	0.4373 (5.88 × 10^−2^)	**0.4807** (7.85 × 10^−2^)
	XGBoost	0.4447 (6.36 × 10^−2^)	0.4486 (6.30 × 10^−2^)	0.4355 (5.77 × 10^−2^)	0.4584 (5.67 × 10^−2^)	**0.4814** (7.10 × 10^−2^)
	DT	0.4039 (3.85 × 10^−2^)	0.3980 (4.30 × 10^−2^)	0.3887 (4.61 × 10^−2^)	0.3967 (5.73 × 10^−2^)	**0.4754** (5.52 × 10^−2^)
CL-Average arousal	RF	0.4477 (4.62 × 10^−2^)	0.4543 (4.60 × 10^−2^)	0.4465 (4.73 × 10^−2^)	0.4717 (6.15 × 10^−2^)	**0.4984** (1.04 × 10^−1^)
	AdaBoost	0.4609 (3.57 × 10^−2^)	0.4506 (3.28 × 10^−2^)	0.4541 (3.62 × 10^−2^)	0.4625 (4.35 × 10^−2^)	**0.5166** (1.30 × 10^−2^)
	GBDT	0.4326 (4.88 × 10^−2^)	0.4408 (4.90 × 10^−2^)	0.4328 (4.73 × 10^−2^)	0.4490 (4.96 × 10^−2^)	**0.5053** (1.40 × 10^−1^)
	XGBoost	0.4336 (5.04 × 10^−2^)	0.4375 (5.03 × 10^−2^)	0.4252 (5.22 × 10^−2^)	0.4510 (6.42 × 10^−2^)	**0.5015** (1.40 × 10^−1^)
	DT	0.4123 (4.24 × 10^−2^)	0.3982 (4.14 × 10^−2^)	0.4027 (4.08 × 10^−2^)	0.3957 (5.56 × 10^−2^)	**0.4879** (9.14 × 10^−2^)
DE-Average valence	RF	0.4293 (3.42 × 10^−2^)	0.4248 (3.03 × 10^−2^)	0.4256 (2.52 × 10^−2^)	0.4389 (2.36 × 10^−2^)	**0.4779** (6.64 × 10^−2^)
	AdaBoost	0.4422 (2.85 × 10^−2^)	0.4482 (2.61 × 10^−2^)	0.4447 (2.90 × 10^−2^)	0.4445 (2.13 × 10^−2^)	**0.4754** (7.42 × 10^−2^)
	GBDT	0.4162 (3.72 × 10^−2^)	0.4211 (3.72 × 10^−2^)	0.4188 (3.34 × 10^−2^)	0.4201 (3.31 × 10^−2^)	**0.4831** (7.32 × 10^−2^)
	XGBoost	0.3885 (3.43 × 10^−2^)	0.3822 (3.56 × 10^−2^)	0.3809 (3.56 × 10^−2^)	0.3924 (3.64 × 10^−2^)	**0.4754** (7.55 × 10^−2^)
	DT	0.4102 (3.61 × 10^−2^)	0.4162 (4.41 × 10^−2^)	0.4031 (3.70 × 10^−2^)	0.3891 (3.87 × 10^−2^)	**0.4738** (6.08 × 10^−2^)
DE-Average arousal	RF	0.4531 (2.52 × 10^−2^)	0.416 (2.74 × 10^−2^)	0.4564 (2.44 × 10^−2^)	0.4635 (1.96 × 10^−2^)	**0.4971** (1.28 × 10^−1^)
	AdaBoost	0.4686 (1.74 × 10^−2^)	0.4703 (1.63 × 10^−2^)	0.4773 (2.09 × 10^−2^)	0.4684 (1.88 × 10^−2^)	**0.5000** (1.50 × 10^−1^)
	GBDT	0.4433 (3.18 × 10^−2^)	0.4379 (3.46 × 10^−2^)	0.4469 (2.69 × 10^−2^)	0.4443 (3.37 × 10^−2^)	**0.4988** (1.49 × 10^−1^)
	XGBoost	0.4037 (3.11 × 10^−2^)	0.3980 (3.86 × 10^−2^)	0.4086 (3.58 × 10^−2^)	0.3977 (4.87 × 10^−2^)	**0.4998** (1.55 × 10^−1^)
	DT	0.4268 (3.59 × 10^−2^)	0.4143 (3.96 × 10^−2^)	0.4053 (3.66 × 10^−2^)	0.3904 (4.03 × 10^−2^)	**0.4953** (1.22 × 10^−1^)

Note: The highest performance metric in each row is in boldface. The terms CL and DE denote the classical feature sets and differential entropy feature sets, respectively. The standard deviation is listed in brackets. The optimal values are shown in boldface.

**Table 5:** Classification performance comparison across different combinations of feature selection methods and machine learning classifiers on the MAHNOB-HCI database.

Performance	Classifier	Feature Selection Method				
		NCA-GFK-CSBS	NCA-GFK-MI	NCA-GFK-RR	NCA-GFK-ERF	MF-DFS
CL-Average valence	RF	0.4333 (8.23 × 10^−2^)	0.4213 (8.41 × 10^−2^)	0.4427 (8.00 × 10^−2^)	0.4421 (8.37 × 10^−2^)	**0.5380** (9.07 × 10^−2^)
	AdaBoost	0.4182 (8.15 × 10^−2^)	0.4317 (7.31 × 10^−2^)	0.4208 (6.62 × 10^−2^)	0.4177 (9.14 × 10^−2^)	**0.5047** (3.56 × 10^−2^)
	GBDT	0.4208 (8.68 × 10^−2^)	0.4015 (8.43 × 10^−2^)	0.4344 (8.38 × 10^−2^)	0.4244 (7.01 × 10^−2^)	**0.5234** (9.56 × 10^−2^)
	XGBoost	0.4208 (6.83 × 10^−2^)	0.4093 (6.31 × 10^−2^)	0.4307 (7.90 × 10^−2^)	0.4375 (6.87 × 10^−2^)	**0.5146** (7.16 × 10^−2^)
	DT	0.3969 (6.60 × 10^−2^)	0.3838 (5.02 × 10^−2^)	0.3974 (6.29 × 10^−2^)	0.3875 (7.06 × 10^−2^)	**0.5078** (7.50 × 10^−2^)
CL-Average arousal	RF	0.4477 (6.68 × 10^−2^)	0.3854 (6.95 × 10^−2^)	0.3771 (7.65 × 10^−2^)	0.4031 (7.37 × 10^−2^)	**0.4755** (6.80 × 10^−2^)
	AdaBoost	0.4609 (4.59 × 10^−2^)	0.3708 (5.22 × 10^−2^)	0.3516 (5.57 × 10^−2^)	0.3948 (6.85 × 10^−2^)	**0.4495** (6.39 × 10^−2^)
	GBDT	0.3719 (6.32 × 10^−2^)	0.3734 (5.92 × 10^−2^)	0.3677 (6.33 × 10^−2^)	0.3828 (8.23 × 10^−2^)	**0.4682** (8.57 × 10^−2^)
	XGBoost	0.3740 (6.56 × 10^−2^)	0.3614 (6.63 × 10^−2^)	0.3521 (6.58 × 10^−2^)	0.3796 (7.25 × 10^−2^)	**0.4646** (4.42 × 10^−2^)
	DT	0.3458 (6.87 × 10^−2^)	0.3802 (6.33 × 10^−2^)	0.3656 (6.72 × 10^−2^)	0.3750 (6.58 × 10^−2^)	**0.4521** (4.54 × 10^−2^)
DE-Average valence	RF	0.4198 (9.21 × 10^−2^)	0.4072 (8.56 × 10^−2^)	0.4026 (8.37 × 10^−2^)	0.4286 (8.12 × 10^−2^)	**0.5120** (9.10 × 10^−2^)
	AdaBoost	0.3932 (9.48 × 10^−2^)	0.3979 (7.47 × 10^−2^)	0.3990 (6.39 × 10^−2^)	0.4073 (7.22 × 10^−2^)	**0.4750** (6.99 × 10^−2^)
	GBDT	0.3911 (9.33 × 10^−2^)	0.3822 (8.44 × 10^−2^)	0.4021 (7.31 × 10^−2^)	0.4062 (8.36 × 10^−2^)	**0.4953** (9.25 × 10^−2^)
	XGBoost	0.4156 (8.50 × 10^−2^)	0.3744 (8.42 × 10^−2^)	0.4177 (7.63 × 10^−2^)	0.4020 (8.73 × 10^−2^)	**0.4859** (6.56 × 10^−2^)
	DT	0.3964 (6.58 × 10^−2^)	0.3713 (8.85 × 10^−2^	0.3818 (6.37 × 10^−2^)	0.3838 (9.31 × 10^−2^)	**0.4745** (6.43 × 10^−2^)
DE-Average arousal	RF	0.4010 (7.33 × 10^−2^)	0.3786 (6.89 × 10^−2^)	0.4208 (7.67 × 10^−2^)	0.4083 (7.95 × 10^−2^)	**0.4661** (5.21 × 10^−2^)
	AdaBoost	0.3776 (6.85 × 10^−2^)	0.3718 (6.17 × 10^−2^)	0.3927 (6.17 × 10^−2^)	0.3870 (6.26 × 10^−2^)	**0.4589** (6.34 × 10^−2^)
	GBDT	0.4208 (8.02 × 10^−2^)	0.3867 (7.26 × 10^−2^)	0.3818 (6.83 × 10^−2^)	0.3630 (5.09 × 10^−2^)	**0.4630** (7.57 × 10^−2^)
	XGBoost	0.3625 (5.32 × 10^−2^)	0.3546 (6.18 × 10^−2^)	0.3724 (6.03 × 10^−2^)	0.3651 (6.55 × 10^−2^)	**0.4578** (4.87 × 10^−2^)
	DT	0.3578 (6.42 × 10^−2^)	0.3635 (6.32 × 10^−2^)	0.3646 (5.43 × 10^−2^)	0.3505 (8.24 × 10^−2^)	**0.4609** (5.00 × 10^−2^)

Note: The highest performance metric in each row is in boldface. The terms CL and DE denote the classical feature sets and differential entropy feature sets, respectively. The standard deviation is listed in brackets. The optimal values are shown in boldface.

**Table 6:** Classification performance comparison across different combinations of feature selection methods and machine learning classifiers on the SEED database.

Performance	Classifier	Feature Selection Method				
		NCA-GFK-CSBS	NCA-GFK-MI	NCA-GFK-RR	NCA-GFK-ERF	MF-DFS
CL-Average valence	RF	0.3326 (3.17 × 10^−2^)	0.3207 (2.48 × 10^−2^)	0.3396 (3.74 × 10^−2^)	0.3478 (4.56 × 10^−2^)	**0.3956** (2.14 × 10^−2^)
	AdaBoost	0.3415 (2.57 × 10^−2^)	0.3385 (3.40 × 10^−2^)	0.3552 (5.68 × 10^−2^)	0.3478 (5.07 × 10^−2^)	**0.3859** (2.35 × 10^−2^)
	GBDT	0.3219 (4.42 × 10^−2^)	0.3381 (2.92 × 10^−2^)	0.3311 (3.27 × 10^−2^)	0.3400 (5.09 × 10^−2^)	**0.3867** (2.04 × 10^−2^)
	XGBoost	0.3178 (3.60 × 10^−2^)	0.3237 (2.72 × 10^−2^)	0.3274 (3.34 × 10^−2^)	0.3363 (4.94 × 10^−2^)	**0.3907** (2.04 × 10^−2^)
	DT	0.3326 (3.64 × 10^−2^)	0.3307 (3.78 × 10^−2^)	0.3370 (5.34 × 10^−2^)	0.3407 (3.24 × 10^−2^)	**0.4037** (1.95 × 10^−2^)
DE-Average valence	RF	0.3463 (4.28 × 10^−2^)	0.3263 (3.70 × 10^−2^)	0.3411 (4.57 × 10^−2^)	0.3415 (3.44 × 10^−2^)	**0.3901** (2.33 × 10^−2^)
	AdaBoost	0.3374 (3.38 × 10^−2^)	0.3189 (3.29 × 10^−2^)	0.3452 (3.30 × 10^−2^)	0.3267 (2.52 × 10^−2^)	**0.3889** (2.68 × 10^−2^)
	GBDT	0.3437 (3.96 × 10^−2^)	0.3278 (3.52 × 10^−2^)	0.3356 (3.13 × 10^−2^)	0.3278 (3.16 × 10^−2^)	**0.3822** (2.61 × 10^−2^)
	XGBoost	0.3452 (4.36 × 10^−2^)	0.3263 (4.09 × 10^−2^)	0.3459 (3.67 × 10^−2^)	0.3315 (3.03 × 10^−2^)	**0.3833** (3.34 × 10^−2^)
	DT	0.3326 (3.35 × 10^−2^)	0.3130 (3.24 × 10^−2^)	0.3330 (3.26 × 10^−2^)	0.3370 (2.45 × 10^−2^)	**0.3974** (2.09 × 10^−2^)

Note: The highest performance metric in each row is in boldface. The terms CL and DE denote the classical feature sets and differential entropy feature sets, respectively. The standard deviation is listed in brackets. The optimal values are shown in boldface.

**Table 7:** Classification accuracy comparison across different machine learning methods in two different feature sets on the DEAP database.

Classifier	DEAP Database			
	Valence		Arousal	
	without Feature Selection	MF-DFS	without Feature Selection	MF-DFS
RF-CL	0.4367	**0.4984**	0.4613	**0.4984**
AdaBoost-CL	0.4215	**0.4959**	0.4719	**0.5166**
GBDT-CL	0.4418	**0.4807**	0.4660	**0.5053**
XGBoost-CL	0.4418	**0.4814**	0.4582	**0.5016**
DT-CL	0.3975	**0.4754**	0.4037	**0.4879**
RF-DE	0.4223	**0.4779**	0.4441	**0.4970**
AdaBoost-DE	0.4307	**0.4754**	0.4541	**0.5000**
GBDT-DE	0.4383	**0.4831**	0.4646	**0.4988**
XGBoost-DE	0.4156	**0.4754**	0.4383	**0.4998**
DT-DE	0.4174	**0.4738**	0.4098	**0.4953**

Note: The highest accuracy in each row for arousal or valence dimension is in boldface. The terms CL and DE indicate the classical and differential entropy features are used for deriving the accuracy.

**Table 8:** Classification accuracy comparison across different machine learning methods in two different feature sets on the MAHNOB-HCI database.

Classifier	MAHNOB-HCI Database			
	Valence		Arousal	
	without Feature Selection	MF-DFS	without Feature Selection	MF-DFS
RF-CL	0.4365	**0.5380**	0.3844	**0.4755**
AdaBoost-CL	0.4135	**0.5047**	0.3922	**0.4495**
GBDT-CL	0.4250	**0.5234**	0.4193	**0.4682**
XGBoost-CL	0.4271	**0.5146**	0.4073	**0.4646**
DT-CL	0.4047	**0.5078**	0.3589	**0.4521**
RF-DE	0.4302	**0.5120**	0.3615	**0.4661**
AdaBoost-DE	0.4104	**0.475**	0.3734	**0.4589**
GBDT-DE	0.4271	**0.4953**	0.3766	**0.4630**
XGBoost-DE	0.4260	**0.4860**	0.3651	**0.4578**
DT-DE	0.4297	**0.4745**	0.3250	**0.4609**

Note: The highest accuracy in each row for arousal or valence dimension is in boldface. The terms CL and DE indicate the classical and differential entropy features are used for deriving the accuracy.

**Table 9:** Classification accuracy comparison across different machine learning methods in two different feature sets on the MAHNOB-HCI database.

Classifier	SEED-CL		SEED-DE	
	Valence			
	without Feature Selection	MF-DFS	without Feature Selection	MF-DFS
RF	0.3685	**0.3956**	0.3393	**0.3901**
AdaBoost	0.3530	**0.3859**	0.3285	**0.3889**
GBDT	0.3477	**0.3867**	0.3315	**0.3822**
XGBoost	0.3555	**0.3907**	0.3211	**0.3833**
DT	0.3470	**0.4037**	0.3241	**0.3974**

Note: The highest accuracy in each row for arousal or valence dimension is in boldface. The terms CL and DE indicate the classical and differential entropy features are used for deriving the accuracy.

## Data Availability

The databases used in this study are publicly available at websites: DEAP: http://www.eecs.qmul.ac.uk/mmv/datasets/deap/, accessed on 20 October 2021; MAHNOB-HCI: https://mahnob-db.eu/hci-tagging/accounts/register/, accessed on 20 October 2021; SEED: https://bcmi.sjtu.edu.cn/~seed/index.html, accessed on 20 October 2021.
